# Antigen Delivery Platforms for Next-Generation Coronavirus Vaccines

**DOI:** 10.3390/vaccines13010030

**Published:** 2024-12-31

**Authors:** Aziz A. Chentoufi, Jeffrey B. Ulmer, Lbachir BenMohamed

**Affiliations:** 1 Laboratory of Cellular and Molecular Immunology, Gavin Herbert Eye Institute, School of Medicine, University of California Irvine, Irvine, CA 92697, USA; aalamich@hs.uci.edu; 2 Department of Vaccines and Immunotherapies, TechImmune, LLC, University Lab Partners, Irvine, CA 92660, USA; jeffreyulmer@techimmune.com; 3 Institute for Immunology, School of Medicine, University of California Irvine, Irvine, CA 92697, USA

**Keywords:** antigen delivery system, antigen delivery platform, SARS-CoV-2, pan-Coronavirus vaccine, mRNA, srRNA, LNP

## Abstract

The COVID-19 pandemic, caused by the Severe Acute Respiratory Syndrome Coronavirus 2 (SARS-CoV-2), is in its sixth year and is being maintained by the inability of current spike-alone-based COVID-19 vaccines to prevent transmission leading to the continuous emergence of variants and sub-variants of concern (VOCs). This underscores the critical need for next-generation broad-spectrum pan-Coronavirus vaccines (pan-CoV vaccine) to break this cycle and end the pandemic. The development of a pan-CoV vaccine offering protection against a wide array of VOCs requires two key elements: (1) identifying protective antigens that are highly conserved between passed, current, and future VOCs; and (2) developing a safe and efficient antigen delivery system for induction of broad-based and long-lasting B- and T-cell immunity. This review will (1) present the current state of antigen delivery platforms involving a multifaceted approach, including bioinformatics, molecular and structural biology, immunology, and advanced computational methods; (2) discuss the challenges facing the development of safe and effective antigen delivery platforms; and (3) highlight the potential of nucleoside-modified mRNA encapsulated in lipid nanoparticles (LNP) as the platform that is well suited to the needs of a next-generation pan-CoV vaccine, such as the ability to induce broad-based immunity and amenable to large-scale manufacturing to safely provide durable protective immunity against current and future Coronavirus threats.

## 1. Introduction

Coronaviruses comprise a vast group of viruses capable of causing a spectrum of illnesses, ranging from mild conditions like the common cold to more serious diseases such as Middle East Respiratory Syndrome (MERS-CoV) and severe acute respiratory syndrome (SARS-CoV) [1,2,3]. The clinical manifestations of infections caused by these viruses are highly variable, spanning from asymptomatic cases to severe disease marked by pneumonia, respiratory distress, and fever. In extreme instances, the disease may advance to acute respiratory distress syndrome (ARDS), septic shock, and death resulting from multi-organ failure. Severe COVID-19, particularly in vulnerable populations like the elderly and individuals with underlying health conditions, has necessitated hospitalization and mechanical ventilation, overwhelming healthcare infrastructures and prompting national lockdowns and large-scale vaccination efforts.

Additionally, the long-term morbidity associated with COVID-19 is significant, with up to 10% of individuals, regardless of initial disease severity, developing long COVID. This chronic condition is characterized by persistent, multisystemic symptoms such as muscle pain, fatigue, and cognitive impairment. The exact mechanisms and immunopathology causing long COVID-19 remain areas of intense investigation. Therefore, a pan-Coronavirus vaccine capable of protecting individuals from disease and reducing the community spread of the virus could help mitigate the burden of disease caused by multiple coronaviruses, including SARS-CoV-2, MERS-CoV, and endemic HCoVs, potentially reducing severe illness, hospitalizations, and long-term complications such as long COVID.

The development of pan-Coronavirus vaccines that protect from the current and future SARS-CoV-2 variants necessitates a multifaceted approach, incorporating molecular and structural biology, immunology, and advanced computational methods. A key to the design of these vaccines is the identification of conserved regions across coronavirus families that can serve as targets for cross-reactive neutralizing antibodies and CD4^+^ and CD8^+^ T-cell immunity [2]. The Spike (S) protein, especially its receptor-binding domain (RBD), has emerged as a primary target for neutralizing antibodies due to its critical role in virus entry into host cells [4]. However, identification of other conserved epitopes remains a significant challenge and an area of active research [2,5,6,7,8]. An effective pan-Coronavirus vaccine, by definition, needs to prevent severe disease and/or infection caused by all viruses within the coronavirus family. The current widely employed SARS-CoV-2 vaccines based solely on the spike glycoprotein were very effective in blunting the severity of the pandemic in its early stages, but waning immunity and antigenic variation between emergent strains have limited their utility. As a result, frequent boosting and updating of the vaccine to better match circulating virus strains are being used to address this limitation. So far, this strategy has not been able to disrupt the transmission cycle; hence, it is not a long-term solution to ending this pandemic or preventing future ones.

This article reviews clinical trial data gathered from public databases, scientific literature, and research announcements up to the current year, 2024. Focus is placed on the current state of antigen delivery platforms best suited for pan-Coronavirus vaccines, emphasizing the challenges and innovations in developing these vaccines that can provide durable immunity against current and future coronavirus threats, evaluating their immunogenicity, efficacy, safety, and cross-reactive potential against various coronavirus strains.

## 2. Antigen Delivery Platforms for Universal Coronavirus Vaccines

Research and development of pan-Coronavirus vaccines have utilized technology platforms such as protein subunits, viral vectors, mRNA, and nanoparticle technologies to deliver the antigen of interest. These platforms have shown promise in eliciting broad and robust immune responses in preclinical models [9,10,11,12,13,14,15,16]. These delivery platforms being explored for pan-Coronavirus vaccine development are at the forefront of immunological research, leveraging cutting-edge science to create vaccines that are not only effective against multiple strains of coronaviruses but can also be rapidly developed and deployed in response to new viral threats. Below we will highlight the delivery platforms that have been proposed for effective pan-Coronavirus vaccines:

### 2.1. Protein Subunit to Deliver Next-Generation PanCoVax Vaccine Candidates

Protein subunit vaccines include pieces (subunits) of the virus (e.g., whole antigens and/or conserved peptides) to stimulate an immune response without introducing the whole virus. These protein-based pan-Coronavirus subunit vaccines are sufficient to teach the immune system to recognize and attack the virus but cannot cause disease since they are only a part of the virus proteome. In addition, immune responses can be targeted to specific viral proteins associated with protection and avoid responses against other proteins that could be ineffectual or deleterious.

A. Protein + adjuvant to deliver next-generation PanCoVax vaccine candidates

Adjuvanted protein-based subunit vaccines constitute a significant category of vaccines. Subunit vaccines face specific challenges compared to other platforms, primarily due to the need for optimization in the production of each protein antigen. Among the most advanced COVID-19 subunit vaccines is Novavax’s NVX-CoV-2373 (marketed as Nuvaxovid), which is produced using insect cells and formulated with the Matrix-M adjuvant, derived from saponin [17]. This vaccine includes the stabilized, full-length spike (S) protein in its prefusion conformation, designed for optimal antigenicity. Similarly, Sanofi-GSK’s vaccine VidPrevtyn Beta contains prefusion-stabilized S protein trimers of SARS-CoV-2, also produced in insect cells and combined with the AS03 adjuvant [18]. Both vaccines have completed human clinical trials and have been authorized for use by the European Union. Recombinant protein vaccines are attractive candidates due to their excellent safety profiles, lack of genome integration risk, absence of live viral elements, and compatibility with immunocompromised individuals. Additionally, they exhibit high production efficiency and stability [17,19,20,21]. In recent studies, seven vaccine formulations containing various combinations of the RBD antigens from SARS-CoV, MERS-CoV, and SARS-CoV-2 XBB.1.5, along with Alum and CpG55.2 adjuvants, were tested in animal models. Mice immunized with the trivalent RBD-based vaccine developed strong antibody responses against all three antigens and efficiently neutralized the corresponding pseudo viruses [22]. To date, only a few protein-based pan-Coronavirus vaccines are being evaluated in clinical trials as detailed in Section 2.5 of this review.

B. Peptides to deliver next-generation PanCoVax vaccine candidates

**Figure 1:**
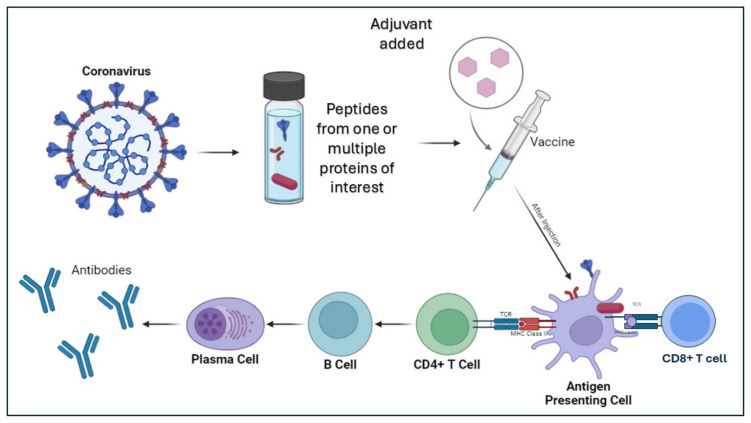
Peptides to deliver next-generation PanCoVax vaccine candidates: A portion of an antigenic protein such as a peptide or polypeptide from coronavirus is used alone or a combination of peptides from different proteins and/or different variants to make a vaccine, potentially with the addition of an adjuvant. After injection of the vaccine, the peptides can be presented to antigen-presenting cells and loaded onto MHC class I for presentation to CD8+ T-cells or through MHC II for presentation to CD4^+^ T cells. This stimulates some B cells to differentiate into plasma cells, which then release antibodies.

Peptide-based vaccines with a carrier also constitute a significant category of vaccines. Through the combination of several peptides, the vaccines increase the likelihood of cross-reactive T- and B-cell responses, as well as create the possibility of using specific peptides to omit immunopathological pro-inflammatory sequences from the vaccine construct and focus the immune response toward critical epitopes (Figure 1). This also allows for flexibility as the peptides can be adjusted to include new epitopes for emerging coronavirus strains [23]. Additionally, the use of peptides rather than the live virus means the vaccine is non-infectious. Since the components of the vaccine are also not redundant with the human genome, there is a significant decrease in the risk of triggering allergic or autoimmune responses [24]. Compared to adjuvanted protein-based subunit vaccines, peptide vaccines have a faster development through automated peptide synthesizers, as well as a fast purification process [23]. The carriers are also straightforward to produce. Both combined allow for standardized mass production of peptide vaccines in an economically feasible manner without bio-contaminants [23,24,25]. When properly adjuvanted, the peptide-based vaccines have been proven to be highly immunogenic. However, the vaccines could also potentially be produced without an adjuvant, utilizing carriers like liposomes. The peptide-based vaccines have also been shown to have greatly improved stability, are water soluble, and have less of a need for cold storage [23,25]. The development is slower than mRNA vaccines, though, due to the time needed for the optimization of peptide epitopes and the carrier. Mosaic-8b from CalTech is a notable example. The mosaic vaccine was engineered using a multivalent protein domain known as SpyCatcher, enabling it to display receptor-binding domains (RBDs) from both human and animal coronaviruses. These RBDs include those from Bat CoV RaTG13, Bat CoV SHC014, Bat CoV Rs4081, Bat CoV RmYN02, Bat CoV Rf1, Bat CoV WIV1, Pangolin CoV Pang17, and SARS-CoV-2, and are presented on a nanoparticle carrier [26]. Additionally, SK Biosciences has advanced efforts toward developing a peptide-based vaccine by fusing RBDs from SARS-CoV-2, SARS-CoV-1, Bat CoV RaTG13, and Bat CoV WIV1 to a carrier protein, I53-50A [26].

### 2.2. Viral Vector to Deliver Next-Generation PanCoVax Vaccine Candidates

**Figure 2:**
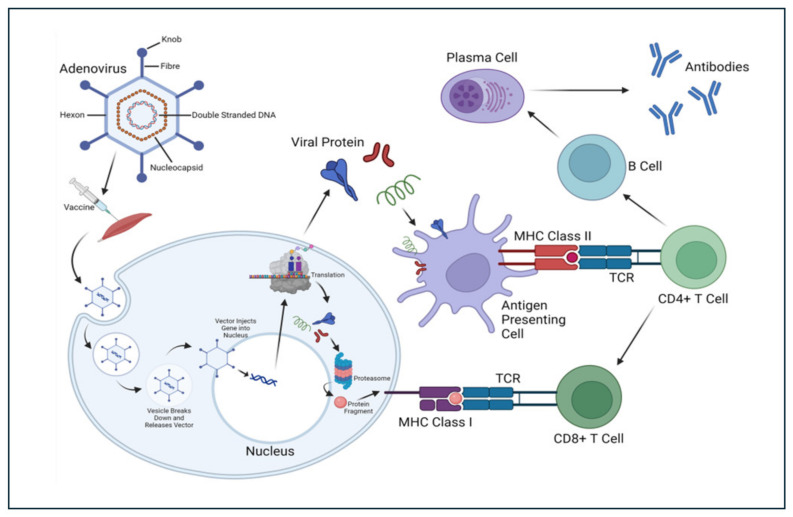
Adenovirus to deliver next-generation PanCoVax vaccine candidates: The adenovirus is non-enveloped and utilizes double-stranded DNA that encodes the antigen of interest from coronavirus. After the construction of the viral vector vaccine, the following steps occur: (1) The vaccine is injected intramuscularly. (2) Muscle cells engulf the cells through endocytosis. (3) The vesicle around the adenovirus breaks down. (4) The adenovirus attaches to the nucleus and transcription begins. (5) The protein is created through translation. After this, the protein peptides can either (a) be loaded onto MHC class I molecules for direct presentation to CD8^+^ T cells or (b) be presented to antigen-presenting cells and loaded onto MHC II for presentation to CD4^+^ T cells, which stimulates some B cells to differentiate into plasma cells, which then release antibodies.

Viral vector vaccines utilize a harmless virus (distinct from the coronavirus) as a vehicle to transport genetic material from the target coronavirus into human cells. Within these cells, the genetic instructions prompt the production of a specific protein from the coronavirus, stimulating the immune system to mount a defense against the virus upon future encounters. This mechanism triggers a potent immune response. These vaccines are known for eliciting a strong and enduring immune reaction, closely resembling the response to a natural viral infection. Viral vectors offer versatility, as they can transport genes encoding multiple proteins, potentially broadening the range of immunity conferred. Among viral vectors, adenovirus stands out, attracting significant attention as a pivotal therapeutic vector due to its status as the first DNA virus to undergo extensive medical exploration (Figure 2). The benefits of adenoviruses as a vaccine platform arise from their advantageous biological properties, including genetic stability, high transduction efficiency, and ease of scalability for large-scale production [27,28]. Adenoviruses are non-enveloped, double-stranded DNA viruses, with genomes typically ranging between 34 and 43 kilobases. In humans, they are commonly associated with mild respiratory and ocular infections [29]. To date, over 150 primate adenoviruses (AdVs) have been identified, many of which are being explored for vaccine development. Unlike certain other viral vectors, adenoviruses do not integrate into the host genome but instead exist in an episomal form [30]. These viruses can be isolated from a variety of species, including humans and animals such as chimpanzees. In humans, approximately 50 adenovirus serotypes have been classified and grouped into seven subtypes (A–G) based on sequence similarities and their ability to induce erythrocyte agglutination [31,32].

A. Adenoviruses to deliver next-generation PanCoVax vaccine candidates

The two primary approaches for developing coronavirus vaccines involve utilizing either entire viruses or genetically engineered vaccine antigens. These vaccine antigens can be administered through various methods [33]. Whole virus vaccines have the potential to elicit a robust immune response, protecting against coronavirus infections. Genetically engineered vaccines, which target specific coronavirus proteins, are extensively employed to enhance vaccine safety and efficacy. Coronavirus proteins, such as Nucleocapsid (N), Spike (S), and Membrane (M), can be delivered through DNA recombinant and viral vector vaccines [34]. A critical aspect of adenovirus-based vaccine development involves the incorporation of a transgene cassette into the adenoviral genome, achieved via homologous recombination or direct cloning [34]. This transgene cassette includes a robust promoter to drive sustained and high-level expression of the introduced genes [34]. In the case of the coronavirus, host proteases cleave the S protein into two subunits, S1 and S2. The Receptor Binding Domain (RBD) within S1 is essential for binding to the Angiotensin Converting Enzyme-2 (ACE2) receptor, while the S2 subunit contains the fusion peptide necessary for membrane fusion and viral entry [35]. The S protein is particularly significant because it contains epitopes that elicit neutralizing antibodies, a focus of many vaccine strategies [36]. Under optimal transfection conditions, high-titer adenoviruses can be produced within 5 days. For example, AstraZeneca and the University of Oxford developed the AZD1222 vaccine that delivers the Coronavirus S gene via an adenoviral vector (ChAdOx1). This vaccine, administered intramuscularly, expresses the S protein in a trimeric prefusion state, essential to its immunogenicity [37]. With eight Phase I, 30 Phase II, and 11 Phase III trials registered worldwide, AZD1222 has undergone extensive clinical testing. Serum Institute of India’s version of AZD1222, Covishield, has completed two Phase II and three Phase III trials. AZD1222 showed a favorable safety profile and a robust immune response in Phase I/II trials among 1090 participants aged 18–55 [38].

B. Adeno-Associated Viruses to deliver next-generation PanCoVax vaccine candidates

Adeno-associated viruses (AAVs) are frequently used as viral vectors for gene therapy. It is well known that AAVs have low immunogenicity and provide sustained gene expression after infection. In previous studies, AAV vector vaccines elicit robust and long-lasting humoral and cellular immune responses in mice and nonhuman primates [39,40,41,42]. COVID-19 vaccine development has tested AAVs extensively. The Spike protein-coding gene from the original SARS-CoV-2 strain was integrated into AAVrh32.33, leading to a long-lasting reduction in neutralizing antibodies. Moreover, this treatment induced a sustained cellular immune response in cynomolgus monkeys, and this response was entirely protective against SARS-CoV-2 [43]. In another study, AAV6 viruses carrying the original SARS-CoV-2 Spike gene were used to generate vaccines that produced higher IgG antibody titers than the inactivated vaccine at a single dose [44].

### 2.3. mRNA to Deliver Next-Generation Universal Coronavirus Vaccine Candidates

mRNA (messenger RNA) vaccines represent a revolutionary approach to vaccine development. Unlike certain traditional vaccines, mRNA vaccines do not use live viruses. Instead, they employ a synthetic strand of mRNA that encodes the instructions for cells to produce a viral protein, such as the Spike (S) protein found in coronaviruses. Once produced by the cells, these proteins stimulate an immune response, teaching the immune system to recognize and combat the virus without being exposed to the disease. mRNA presents several advantages such as rapid development: mRNA vaccines can be developed swiftly, as they require only the genetic sequence of the virus; high flexibility: mRNA vaccines can be easily adapted to target new mutations or multiple strains by tweaking the mRNA sequence and highly safe, as no live virus is used, there is little to no risk of causing disease in the vaccinated individual [2,12,45,46,47,48].

Various types of mRNA have been suggested and employed for vaccine delivery. They have been categorized based on their mode of action into two types: conventional mRNA and self-amplifying (or self-replicating) mRNA vaccines [49,50,51]. Currently, there are five types of RNA vaccines: conventional and base-modified, non-amplifying mRNA, which incorporate chemically modified nucleotides and have been widely used for COVID-19 vaccines; self-replicating RNA (srRNA), which is based on an engineered viral genome but devoid of viral structural protein genes; Circular mRNA (circRNA) that is a class of single-stranded RNAs with covalently closed structures, which protects it from exonuclease-mediated degradation [52]; and thermostable mRNA, which uses lyophilizable, thermostable nanostructured lipid carrier to effectively deliver mRNA [53,54].

**Figure 3:**
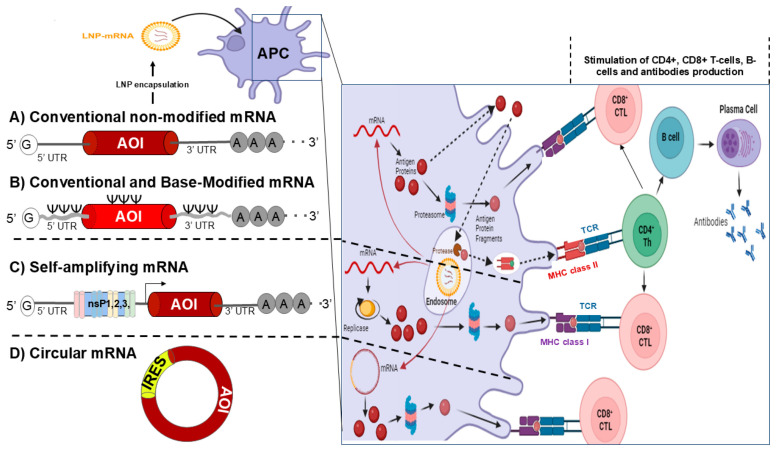
Pan-Coronavirus mRNA-LNP based vaccines: Pan-Coronavirus vaccines involve the use of an mRNA transcript that encodes an antigen and LNP. Linear non-replicating mRNAs include a sequence for antigens of interest (AOI, such as the Spike, Nucleocapsid, and other conserved proteins) flanked by 5′ and 3′ untranslated regions (UTRs), a cap structure at the 5′ end, and a poly(A) tail at the 3′ end. Depending on whether modified or native nucleosides are used during in vitro transcription, either (A) unmodified or (B) modified mRNAs are produced; (C) Self-amplifying RNA (saRNA) has a similar sequence organization but additionally includes (1) a sequence encoding four non-structural proteins (NSP1–4) that form a replicase to amplify the saRNA, and (2) a viral-origin subgenomic promoter (black arrow) that initiates antigen transcription; (D) Circular RNA (circRNA)-based vaccines consists of a covalently closed single-stranded RNA containing the sequence of antigens of interest and an internal ribosome entry site (IRES) to initiate antigen translation. The antigens of interest are produced endogenously by the antigen-presenting cells (APCs) translational machinery (red circles), degraded by proteasomes (pink circles), and presented on major histocompatibility complex (MHC) class I molecules (pink circles), leading to a specific CD8+ cytotoxic T cell response against coronaviruses variant of concern. Additionally, the antigen protein can be exported from the cell, endocytosed by the same or another APC, degraded by endosomal proteases, and presented on MHC II molecules, resulting in a CD4^+^ helper T cell response. Immunization progresses with CD4^+^ helper T cells aid in the activation of B cells to produce anti-coronavirus-specific antibodies and neutralizing antibodies. CD8^+^ cytotoxic T cells specifically target and eliminate coronavirus-infected cells.

The advantages of conventional and base-modified mRNA vaccines are: (1) highly effective in protein translation, (2) non-infectious and non-integrating, making them low-risk for infection and/or genetic alteration, (3) capable of being rapidly produced, and (4) adaptable to various pathogens. However, conventional, and base-modified mRNA vaccines require cold storage (−20 °C to −70 °C) to maintain their stability, and mRNA degrades quickly in cells (6–12 h), which limits the duration of antigen expression. mRNA can be non-amplifying, consisting of straightforward mRNA sequences that directly encode the target antigen, which offers direct translation into protein antigens without the need for viral replicase components. However, antigen expression duration is limited (1–3 days), meaning they will potentially require booster doses. Interestingly, self-replicating (srRNA)-based vaccines include both the mRNA for the target antigen and viral replicase machinery, which allow self-replication within cells, leading to longer antigen expression (1–2 months) and achieving effective immune response with lower doses and leading to a stronger and more sustained immune response. srRNA-based vaccines also require cold storage (−20 °C to −70 °C) for stability. Their larger size (10–15 kb) complicates encapsulation/delivery and manufacturing, which may introduce additional safety concerns and regulatory hurdles. Recently, circular mRNA (circRNA)-based vaccines are presented as an alternative to linear conventional and base-modified mRNA; instead, they have a closed-ring structure. The advantages of this type of vaccine are that their circular mRNA is resistant to exonucleases, leading to increased stability and longer half-life in cells (1–3 weeks), which offers a prolonged duration of antigen expression (1–3 months). Importantly, circular mRNA does not require cold storage to remain stable; they can be stable at room temperature. To overcome the mRNA stability in high temperatures, thermostable mRNA vaccines were engineered with specific sequences and formulations that maintain the mRNA’s integrity and function in higher temperatures. Aside from their ability to withstand high temperatures, some other advantages of this vaccine are that they are low risk because they are non-infectious and non-integrating, and they can be rapidly produced and distributed in response to outbreaks, especially in regions without cold chain infrastructure (Table 1 and Figure 3).

Various methods have been designed for delivering mRNA, tailored to specific applications. The most common approaches include liposomes, electroporation, gene guns, viral vectors, penetrating peptides, and polymers. Although naked, unformulated mRNA is expressed in cells; its cellular uptake is generally very low, around 0.01% [55,62,63,64]. It is important to note that many of these delivery methods are primarily suitable for in vitro use. For instance, liposomes, which were the first delivery method employed in Nano pharmaceuticals, serve as versatile carriers capable of transporting both hydrophobic and hydrophilic substances, including proteins, nucleic acids, and small molecules [65]. Liposomes usually range from 20 to 1000 nm in size and consist of one or more lipid bilayers encapsulating aqueous and hydrophobic compartments. Studies have shown a relationship between liposome size and uptake by macrophages in vivo, emphasizing the need for precise size control in liposome-based delivery systems [66]. This necessitates careful measurement of liposome size for accurate delivery purposes [67]. Currently, lipid nanoparticles (LNPs) are the most commonly used method for therapeutic mRNA delivery. LNPs consist of positively charged cationic and ionizable lipids that are complex with the negatively charged mRNA, thereby enhancing cellular uptake, and facilitating endosomal escape [68,69]. Despite being less immunogenic and toxic compared to viral vectors, LNPs still exhibit some cytotoxicity, which can be influenced by lipid composition or PEG modifications. This cytotoxicity may lead to membrane damage or vacuolization of cytoplasm and could potentially disrupt crucial cellular pathways and cell cycle stages. To mitigate these effects, new lipids are being developed to enhance delivery efficiency while reducing residence time in tissues and cytotoxicity. However, producing LNPs at a large scale can be challenging, but solid lipid nanoparticles and nanostructured lipid carriers have been developed to address these limitations, offering improved stability, sterilizability, and bioavailability compared to traditional LNPs [65].

A. Conventional modified RNA (mRNA) to deliver next-generation PanCoVax vaccine candidates

Conventional mRNA vaccines utilize cellular machinery to translate the given protein (Figure 3A,B). In contrast, self-amplifying mRNA vaccines include additional coding for accessory proteins, such as RNA-dependent RNA polymerase, capping enzymes, and proteases, which facilitate self-replication alongside the target RNA (Figure 3C) [57,70,71,72]. Advantages of the mRNA technology include but are not limited to: (1) expression of antigens in situ results in the native conformation of protein antigens; (2) it mimics a virus infection, thereby inducing broad-based immunity without the need for a virus vector; and (**3**) generic methods of synthetic production, purification, and characterization of mRNA products lead to faster discovery, development, and implementation of vaccines. This last attribute is critically important for infectious disease outbreaks like COVID-19, enabling a much faster response than possible. Currently, two major conventional mRNA-based vaccines developed by Moderna and Pfizer have been widely used during the COVID-19 pandemic [19,73,74]. Both vaccines employ a similar principle, utilizing mRNA encapsulated in lipid nanoparticles (LNPs) to encode the Spike protein. These vaccines have been administered to billions of individuals and have effectively protected against moderate and symptomatic SARS-CoV-2 infection. It should be noted, however, that immunity induced by these necessitates the use of booster vaccines [73,75]. Currently marketed mRNA-based vaccines against COVID-19 have shown some caveats and potential side effects [76]. The most significant shortcomings are short-lived protection and reduced efficacy towards variants of concern [77]. However, they were able to induce protective antibodies and CD4+ and CD8+ T cells for only up to 6 months post-vaccination. We and others believe that a good coronavirus vaccine should trigger not only antibody response but also strong and long-lasting CD4+ and CD8+ T-cell immune responses [77]. Rare but serious adverse events such as the vascular system, myocardial infarction, stroke, and pulmonary embolism were specifically associated with mRNA vaccines, which has triggered some public concerns and vaccine hesitancy [77,78]. In general terms, complications from SARS-CoV-2 infection outweigh the risk of suffering these rare adverse effects following mRNA- based vaccination, and billions of individuals around the globe have received these mRNA vaccines, which helped significantly in the reduction of the burden of the COVID-19 pandemic.

B. Design and production of next-generation “pre-emptive” PanCoVax vaccine candidates

We have created PanCoVax vaccine constructs containing 10 bioinformatically designed consensus sequences of SARS-CoV-2 antigenic regions that are carefully selected as being highly conserved across all human and animal CoV and are strongly immunogenic (“PanCoVax” candidates) [79]. Multiple lines of evidence demonstrate (1) Three out of these 10 T-cell PanCoVax vaccine candidates alone conferred protection in the hamster model against infection with the SARS-CoV-2 Delta variant, as measured by reduced weight loss and virus replication. (2) When combined with the Spike antigen, these T cell PanCoVax vaccine candidates provided an additive or synergistic protective efficacy against infection with Delta and re-infection with Omicron, by promoting cross-protective CD4+ and CD8+ T cells (against non-Spike antigens) and broadly neutralizing antibodies (nAb, against Spike), suggesting that multi-antigen PanCoVax vaccines have the potential to terminate infection before transmission and cross-protect against new variants, other HCoV, and future CoV zoonoses [79]. This T-cell immunity would support efficacy in individuals with antibody defects and generate longer-lasting protection due to the inherent durability of T helper cell memory. We demonstrated that delivery of these multi-antigen PanCoVax vaccines boosts durable frontline mucosal immunity by enhancing: (1) the frequency and function of tissue-resident cross-reactive CD4+ and CD8+ TRM cells in the lungs; and (2) cross-reactive IgA/IgG antibodies levels in alveolar lavage that limited viral replication to abort infection in the earliest stages and block transmission [79,80,81].

A safe and effective PanCoVax vaccine inducing systemic and mucosal anti-viral B- and T-cell immunity against the emergence and rapid expansion of SARS-CoV-2 variants is currently not available [82]. For the past 5 years, we have made significant progress in the development of the multi-antigen PanCoVax vaccine, identifying potential human B-cell, CD4^+^ T cells, and CD8^+^ T cell antigens and epitopes from the whole SARS-CoV-2 genome sequences spanning the virus’s multi-stage lifecycle. We used the sequences of the first SARS-CoV-2 strain immediately after it became publicly available in early January 2020 [83,84,85,86,87,88,89]. Using highly conserved large sequences comprised of 10 antigens, we designed 10 PanCoVax vaccine candidates. The use of large protein antigenic sequences will provide broader coverage in the human population by allowing multiple CD4^+^ and CD8+ T-cell epitopes to be presented by multiple HLA types and obviates the need to identify specific T-cell epitopes. In addition, targeting CD4^+^ and CD8^+^ T cells to conserved regions that are subdominant in natural infection and/or that are not targeted by any currently licensed vaccines, is expected to provide an additional “layer” of protective immunity not currently seen in the infected and vaccinated population. Thus, the use of highly conserved antigenic regions will generate cross-reactive immunity that is expected to protect against new VOCs and zoonoses. The use of established vaccine platforms such as base-modified mRNA encapsulated in lipid nanoparticles (LNP) for which toxicity, safety, scalability, and manufacturing are well-described substantially de-risks the program. The mRNA/LNP-based multi-antigen PanCoVax vaccine candidates incorporate common B-cell, CD4^+^, and CD8^+^ T-cell antigens that are conserved in human SARS-CoV and animal SARS-CoV-like strains.

Our data demonstrate that the first three mRNA/LNP-based PanCoVax vaccine candidates (i.e., NSP-2, NSP14, and Nucleocapsid) induced protective CD4+ and CD8+ T-cell-mediated protection against infection with the Delta variant [2]. We selected the mRNA/LNP-based delivery system because it facilitates the presentation of antigens to both MHC-I (endogenous) and MHC-II (exogenous) pathways via direct- and cross-presentation [90,91,92,93,94,95,96,97,98,99,100]. When administered to golden Syrian hamsters in combination with the Spike protein as nucleoside-modified mRNA encapsulated in lipid nanoparticles (LNP)-forming a combined mRNA/LNP-based pan-CoV vaccine, they: (i) induced high frequencies of lung-resident antigen-specific CXCR5+CD4+ T follicular helper (TFH) cells, GzmB+CD4+ and GzmB+CD8+ cytotoxic T cells (TCYT), and CD69+IFN-γ+TNFα+CD4+ and CD69+IFN-γ+TNFα+CD8+ effector T cells (TEFF); and (ii) reduced viral load and COVID-19-like symptoms caused by various VOCs, including the highly pathogenic B.1.617.2 Delta variant and the highly transmissible, heavily Spike-mutated XBB1.5 Omicron sub-variant. This combined mRNA/LNP vaccine can provide broader cross-protective immunity against emerging, highly mutated, and pathogenic VOCs [1,2]. These results demonstrate the successful design and construction of an mRNA/LNP-based multi-antigen PanCoVax vaccine candidate and confirm the utility of previous mRNA/LNP-based vaccines in other systems [2].

Besides our mRNA/LNP-based PanCoVax vaccine, many others have developed and produced different next-generation pancoronavirus vaccines using different platforms and targeted antigens. Some of these candidates have demonstrated high capacity to induce broad-spectrum neutralizing antibodies and T-cell responses, have been successful in preclinical animal study, and some are in clinical trials (See Section 2.5 of this Review).

C. Self-replicating RNA (srRNA) vaccine platforms to deliver next-generation PanCoVax vaccine candidates

Compared to conventional mRNA, the srRNA vaccine delivery platform offers several additional and attractive features that make this vaccine delivery system well-suited for application to our next-generation, broad-spectrum Pan-CoVax vaccine project. These attributes include a longer duration of transgene expression and an enhanced adjuvant effect leading to more potent antigen-specific adaptive immunity, particularly T-cell responses, which is a key feature of our strategy to induce strong, cross-strain T-cell responses and thereby provide broad protection (Figure 3C). In addition, there is a higher potential for co-expressing multiple transgenes from the same srRNA molecule [57]. Moreover, due to their amplification ability, srRNA vaccines could be effective at lower RNA doses, which in turn would lead to higher manufacturing volumes, lower costs, and an improved therapeutic index compared to conventional mRNA vaccines [71]. Potent antigen-specific B- and T-cell responses can be induced by srRNA at doses as low as picograms in an animal model [57]. The best example of the srRNA pan coronavirus vaccine is being developed by Gritstone, encoding viral antigens, including both Spike protein and conserved non-Spike CD8 epitopes aimed at eliciting T-cell responses [58]. Arcturus’ self-amplifying COVID-19 mRNA vaccine candidate (ARCT-154) employs a replicon based on the Venezuelan equine encephalitis virus (VEEV), where the VEEV structural proteins have been replaced with the stabilized, mutation-full-length ancestral Spike protein of SARS-CoV-2. Phase 1/2/3a/3b trials were conducted and demonstrated that two doses of ARCT-154 were well tolerated and immunogenic at the time the delta (B.1.617.2) variant was circulating. [101]. ARCT-154 has been announced to meet the primary efficacy endpoint in the Phase 3 study and was recently approved by regulators in Japan [102].

D. Circular mRNA vaccine platforms to deliver next-generation PanCoVax vaccine candidates

Although mRNA has shown promising efficacy in infectious disease and cancer vaccines, its application is hindered by challenges related to expression duration, stability, and immunogenicity. mRNA has a short lifespan and is quickly degraded by intracellular enzymes and the immune system, typically persisting for only 1–3 days [103]. Consequently, mRNA vaccines often result in relatively low levels of antigen expression, necessitating repeated inoculations to achieve sufficient immune responses. However, a new class of therapeutic mRNAs, circular RNAs (circRNAs), is emerging and may help overcome some of the drawbacks associated with linear mRNAs. CircRNAs offer enhanced stability and reduced autoimmunity, potentially providing a solution to these challenges [104,105]. In contrast to linear mRNA, circRNA boasts a notably high stability attributed to its covalently closed ring structure, which shields it from degradation mediated by exonucleases (Figure 3D) [59]. Studies have indicated that circRNAs possess a median half-life that is at least 2.5 times longer than their linear mRNA counterparts in mammalian cells [106,107]. This enhanced stability allows for protein translation from circRNA to persist for nearly a week, resulting in a significantly higher protein yield compared to linear mRNA, often increased by hundreds of times [103,104,107]. Therefore, CircRNA represents a superior alternative for sustained protein expression, providing enhanced antigenic stimulation and enabling robust antigen production for up to a week [103]. CircRNA vaccines hold promise for enhancing the stability and immunogenicity of mRNA vaccines due to their closed-loop structure, which confers increased stability both in vitro and in vivo, thereby facilitating extended expression of target genes [103]. Pancoronavirus circRNA^RBD^ vaccines have been shown to elicit high levels of broad-spectrum neutralizing antibodies against Delta, Omicron, and against the current VOCs and long-lasting protective immunity against SARS-CoV-2 in mice and rhesus macaques [108].

E. Thermostable mRNA vaccine platforms to deliver next-generation

PanCoVax vaccine candidates: One of the major obstacles facing current coronavirus vaccines is the challenge of widespread distribution during a pandemic [4,109,110,111]. Both authorized vaccines require deep cold chain storage (−70 °C for Pfizer/BioNtech and −20 °C for Moderna’s SARS-CoV-2 RNA vaccines). Even frozen shipping and storage under standard freezer conditions pose difficulties, especially in areas with limited medical infrastructure [112,113,114]. The instability of RNA vaccines presents a significant challenge, with the underlying physicochemical causes still lacking comprehensive research or understanding. However, some aspects are evident. Firstly, RNA vaccine molecules are prone to cleavage by common ribonucleases (RNases), despite attempts to engineer stabilization. Secondly, because of its size, negative charge, and hydrophilic nature, RNA faces difficulties in traversing cell membranes to reach target cells post-injection. Hence, formulations for RNA delivery become essential to stabilize and shield RNA molecules from degradation [55,63,115]. The primary approach employed for administering RNA vaccines, encompassing most SARS-CoV-2 vaccines under clinical investigation to date, revolves around the use of lipid nanoparticle (LNP) delivery systems, where the negatively charged RNA molecule is enclosed within an intricate lipid framework, yielding RNA/LNP complexes typically sized between 70 and 100 nm in diameter. These complexes serve to shield the RNA from degradation by RNases and facilitate successful endocytosis by the cell [116,117]. Nevertheless, both the stability of RNA and LNP remain problematic [114], as indicated by sensitivity to freezing temperatures, leading to adverse effects on their colloidal stability upon freeze/thaw cycles. Consequently, several alternative lipid-based delivery systems have been suggested and advanced for administering RNA vaccines [60,111,114,118,119]. However, a critical need remains for an effective and thermostable RNA vaccine delivery system. Research has demonstrated the effectiveness of a lyophilizable, thermostable nanostructured lipid carrier in delivering both mRNA- and self-replicating RNA-based vaccines via intramuscular injection. When stored refrigerated, the liquid nanostructured lipid carrier alone remains stable for at least 1 year. Additionally, lyophilized nanostructured lipid carrier-RNA complexes have been shown to maintain their biophysical properties and capacity to induce protein expression in vivo for at least 8 months at room temperature and at least 21 months under refrigerated conditions.

### 2.4. Nanoparticle Technologies to Deliver Next-Generation PanCoVax Vaccine Candidates

Classical liposomes, as an early iteration of lipid nanoparticles (LNPs), have served as versatile nanocarriers capable of delivering both hydrophobic and hydrophilic therapeutic agents. However, recent advancements have focused on addressing the limitations of traditional lipid nanoparticles by enhancing their stability, increasing drug-loading capacity, and offering better-controlled release profiles. These improvements also aim to reduce the potential for drug leakage, thanks to the solid lipid core that allows for more efficient drug encapsulation while maintaining excellent biocompatibility and biodegradability. This has led to the development of subsequent generations of LNPs, such as solid lipid nanoparticles (SLNPs), nanostructured lipid carriers (NLCs), and cationic lipid-nucleic acid complexes [120]. These newer LNPs exhibit more complex structures and greater physical stability compared to older systems like liposomes or simple emulsions [65,121]. A key advantage of LNPs is their ability to form spontaneously or self-assemble, driven by interactions between charged groups in the lipid mixture and charged nucleic acids. Nanoparticle vaccines use tiny, engineered particles that can mimic the virus’s structure or be used as vehicle platforms of RNA and proteins. These nanoparticles can display multiple copies of viral RNAs and proteins enhancing the immune system’s ability to recognize and remember the virus. The delivery of RNA and repetitive display of the antigen can trigger a stronger immune response. Nanoparticles can be engineered to improve the stability of the vaccine and enhance delivery to the immune system.

A. Self-Assembling Protein Nanoparticles are successful in pre-clinical animal models but unsuccessful in clinical vaccine trials against infectious diseases

Self-Assembling Protein Nanoparticles (SAPNs) were discovered back in the 2000s and actively developed from 2006 to 2024 as a platform for vaccine delivery due to their ability to self-organize into structures with a potential to induce or boost B and T cell responses [96,122,123,124]. SAPNs consisting of proteins as building blocks are designed using computer modeling [96,122,123,124]. The self-assembling properties of nanoparticles with regular polyhedral symmetry and a diameter of 16 nm have been demonstrated using structures based on a single protein chain [96,122,123,124]. An oligomerization domain comprises two coiled coils joined by a short linker segment for each protein chain [96,122,123,124]. As proteins dissolve in aqueous solutions, they form nanoparticles of 20 nm diameter [90,98]. Researchers in 2015 and 2017 reported developing a malaria vaccine based on SAPNs that displays *Plasmodium falciparum*-derived B- and T-cell epitopes [90,98]. When assembled into SAPNs, the resulting protein sequence induced strong, long-lived, and protective immune responses against the parasite infection in a mouse model [90,98].

However, to the best of our knowledge, although this SAPNs platform has been extensively studied since 2006 [90,96,98,122,123,124], (1) there are no reported peer-reviewed studies demonstrating the superiority of this SAPN platform compared to the most recent mRNA/LNP platform when it comes to safety, immunogenicity, and protective efficacy in animal models and against any infectious diseases, including SARS-CoV-2; and (2) moreover, there are no reported successful clinical trials against any infectious diseases, including SARS-CoV-2, using the SAPNs technology described above. Thus, although this SAPNs-based malaria vaccine candidate was successful in pre-clinical studies in animal models [90,98], a subsequent SAPNs-based malaria vaccine clinical trial appears to have been terminated for reasons that remain to be determined.

Therefore, the above SAPN vaccine delivery platform appears to still require further development to create a safe, immunogenic, and protective antigen delivery platform that can be adapted to the clinic. As discussed by many other research groups [125,126,127,128], several challenges might be associated with using this SAPN platform for vaccine delivery in the clinic: (1) Compared to the mRNA/LNP platform, the SAPN platform used selected expression systems that requires achieving consistent assembly and stability of SAPNs that are crucial for ensuring effective vaccine delivery and prolonged shelf life [129]; (2) Compared to the mRNA/LNP platform, potential spacial and conformational issues and protein display, orientation, denaturation, and aggregation could affect the efficacy of SAPNs-based vaccines [129]; (3) Compared to the mRNA/LNP platform, consistent and efficient load multiple antigens and control release from the SAPNs platform for effective vaccine delivery remains a challenge [130]. In addition, limitations in the amount of protein antigen that can be loaded on a single SAPN make this delivery system not compatible with combination vaccines that use several protein antigens [126]; (4) Compared to the mRNA/LNP platform, SAPNs efficacy to target and activate the desired effector, helper and follicular memory CD4+ and CD8+ T-cells and particularly neutralizing antibodies may not be consistently achieved by the SAPN system without adding built-in adjuvants that may present toxicity [127]; (5) Compared to the mRNA/LNP platform, one must ensure that SAPNs do not induce unwanted immune responses or toxicity, particularly when built-in adjuvants are added, a critical point for safe use of this SAPNs platform in the clinic [127]; and (6) Compared to the mRNA/LNP technology platform, regulatory requirements for SAPNs-based vaccines may involve complex testing and compliance with standards [125,128].

B. Lipide Nanoparticles-based vaccines to deliver next-generation mRNA-based PanCoVax vaccine candidates

Several benefits can be gained from using lipid nanoparticles (LNPs) as delivery system for next-generation mRNA-based PanCoVax vaccine candidates. Among these benefits are lower toxicity, improved immunity, ease of production, and scalability [69,131,132]. LNPs protect the mRNA from degradation and deliver it to antigen-presenting cells. The instability of mRNAs and their susceptibility to degradation by nucleases and self-hydrolysis make LNPs crucial for encapsulating mRNAs, protecting them from external ribonucleases, and aiding in vivo and intracellular delivery [116,131,133]. LNPs are also non-toxic and biodegradable. Therefore, they are an ideal delivery system for mRNA vaccines. While most research on mRNA/LNP vaccines focus on the nucleic acid components, the significance of LNP carriers has been less explored and their composition and biophysical properties have yet to be fully understood in relation to the vaccine’s immune, protective, and anti-inflammatory effects.

In order for LNPs to be stable and efficient, four essential components need to be present: ionizable lipids, which are essential for mRNA binding; helper lipids, which enhance LNP stability and delivery efficiency; cholesterol, which maintains structural integrity; and PEGylation with poly (ethylene glycol) (PEG) or derivatives, which minimizes immune detection and improves circulation [131]. In addition to their simple formulation, self-assembly ability, biocompatibility, and ability to carry large amounts of payloads, these lipids also have several other advantages over others, such as their ability to self-assemble. In a physiological pH range, ionizable lipids remain neutral; with an acidic pH range. However, they become positively charged, forming complexes with negatively charged mRNA and facilitating their release into the cell [134]. Through cell membrane absorption and subsequent endocytosis, the encapsulated mRNA is delivered to the cell. In the presence of negatively charged cell membranes and positively charged LNPs, electrostatic interactions help release mRNA [135]. In vivo delivery of mRNA is rendered efficient with high encapsulation, stability, and pH-sensitivity of mRNA through the neutralization of LNP charge by cellular anionic lipids upon cellular entry, thus enhancing the encapsulation, stability, and pH-sensitivity of mRNA upon cellular entry. LNP size is important for delivery, particularly for larger RNA sequences such as srRNA. However, how this impacts the mRNA delivery is still to be understood. Our group has done a comparative evaluation of different LNPs to determine optimal LNP characteristics for mRNA-based vaccine outcomes and investigated the efficacy of four different LNPs (LNP-1, LNP-2, LNP-3, LNP-4) composed of different phospholipid compositions and ionizable lipid amounts and have shown that the LNP size and physicochemical composition of the lipids affect significantly the degree of protection obtained with mRNA-LNP vaccines against SARS-CoV-2 challenge (unpublished data).

Currently, several mRNA-based pancoronavirus vaccines against coronavirus are utilizig different LNP formulations, including Pfizer (BNT162b4) and Arcturus (ARCT-154), Gritstone bio (GRT-R910) and University of California Irvine/TechImmune LLC (un-named). Pfizer-BioNtech uses an ionizable cationic lipid called ALC-0315, licensed from Acuitas, with nanoparticles comprising ALC-0315, ALC-0159, 1,2-stearoyl-sn-glycerol-3-phosphocholine (DSPC), and cholesterol, where ALC-0159 is a PEGylated lipid [136] and University of California Irvine/TechImmune LLC are using of the shelf Cytiva proprietary LNP (unpublished data).

### 2.5. Current Delivery Platforms of Pan-Coronavirus Vaccines in Clinical Trials

The imperative for a universal coronavirus vaccine is driven by the continuous threat posed by multiple coronaviruses affecting humans, such as SARS-CoV-2, Middle Eastern respiratory syndrome coronavirus (MERS-CoV), and the four endemic human coronaviruses (HCoVs OC43, HKU1, 229E, and NL63) [137,138,139]. Prophylactic vaccines could significantly reduce the morbidity attributed to these pathogens. Although still in the early stages, clinical trials have provided encouraging data on the safety and potential efficacy of pan-Coronavirus vaccines [140,141,142]. Some candidates have demonstrated the capacity to induce broad-spectrum neutralizing antibodies and T-cell responses, suggesting cross-protection against multiple coronavirus strains [143,144]. Staying ahead of virus mutations is a significant challenge for vaccine developers. Developing pan-Coronavirus vaccines is difficult due to the diverse ways coronaviruses use entry receptors. SARS-CoV-1, SARS-CoV-2, and NL63 utilize ACE2, while MERS uses DDP4, and OC43 and HKU1 utilize 9-O-acetylated sialic acid. Additionally, 229E employs the aminopeptidase N receptor. Neutralizing antibodies block the interaction between the virus’s receptor-binding domain and the host cell entry receptor. A pan-Coronavirus vaccine would, therefore, require epitopes that bind universally to all Coronavirus entry receptors and is still to be developed. T cells are more cross-reactive than B cells since they recognize peptide sequences rather than complex structural epitopes in major histocompatibility complexes (MHC). Furthermore, T cells target conserved viral proteins, whereas B cells target surface and structural proteins accessible to antibodies on extracellular viruses. T-cell immunity is highly adaptable, underscoring its ability to recognize and respond to various pathogenic challenges, including those posed by diverse Coronaviruses [145]. Most SARS-CoV-2-specific T cells have been demonstrated to be at least pan-variant reactive, meaning they can recognize epitopes that are retained in currently circulating viruses [87,146,147]. As such, apart from a few key exceptions, instances of T-cell escape are rare. This suggests that T-cell-mediated immunity may play a crucial role in providing broad protection against various SARS-CoV-2 variants, contributing to the effectiveness of pan-Coronavirus vaccine strategies [148,149,150]. Currently, few clinical trials aim to protect against different subgroups of coronaviruses, using different delivery systems, and giving promising results in Phase1/2 trials (Table 2):

***Pfizer BNT162b4:*** Pfizer’s vaccine candidate BNT162b4 (an mRNA vaccine encoding segments of the N and M proteins, and short segments from the ORF1ab polyprotein), developed to enhance T-cell responses against conserved non-spike antigens of SARS-CoV-2, encodes conserved, immunogenic segments of the nucleocapsid, membrane, and ORF1ab proteins targeting diverse HLA alleles. BNT162b4 elicits robust CD4+ and CD8+ T-cell responses to a wide array of epitopes in preclinical animal models while preserving Spike-specific immunity. Notably, BNT162b4 has been shown to protect animal models from severe disease and reduce viral loads following infection with various SARS-CoV-2 variants [151].

***Gritstone GRT-R910:*** GRT-R910 is an investigational vaccine that was designed to enhance immunogenicity and protective efficacy against the current and future SARS-CoV-2 VOC. A randomized, double-blinded Phase 2b study has been conducted to assess the efficacy, safety, and immunogenicity of Gritstone bio’s next-generation COVID-19 vaccine candidate compared to an approved COVID-19 vaccine. Gritstone’s vaccine incorporates a self-replicating mRNA, encoding viral antigens, including both Spike protein (as seen in first-generation COVID-19 vaccines) and conserved non-Spike CD8 epitopes aimed at eliciting T-cell responses [58].

***OVX033 from Osivax:*** A protein subunit vaccine, aiming to protect against sarbecoviruses, the subgroup of coronaviruses from which SARS viruses come. The vaccine contains the full-length nucleocapsid antigen of SARS-CoV-2 which is genetically fused to the self-assembling sequence OVX313, which is a 55-amino acids sequence, hybrid of the C-terminal fragments of two avian C4bp α-chain sequences, that naturally oligomerizes into heptamers. The OVX033 aims to target the nucleocapsid (N) protein within SARS-CoV-2, which is highly conserved among the Sarbecoviruses [159]. OVX033 was tested either unadjuvanted or formulated with a squalene-in-water emulsion containing cholesterol and QS21 saponin [159]. After evaluating the efficiency of the OVX033 vaccine using a hamster model of SARS-CoV-2 infection, the vaccine proved effective against three different SARS-CoV-2 variants of concern as seen through a significant decrease in weight loss, lung viral loads, inflammation, lymphoplasmacytic perivascular infiltration, and pneumonia incidence. The vaccine also showed improved immunogenicity as T-cell responses were triggered within the hamsters due to the vaccine. These sufficient results supported further evaluation of the vaccine within human trials. Currently, participants are being recruited in Paris to test the safety and immunogenicity of three dosages—they are aiming for 48 participants.

***PanCoV from LinkInVax, developed by INSERM:*** A protein subunit vaccine aiming at sarbecoviruses. It was developed by INSERM, the French national health agency similar to the US NIH. The vaccine targets the RBD of the SARS-CoV-2 spike protein to the CD40 receptor and includes T- and B-cell epitopes spanning sequences from S and nucleocapsid (N) proteins from SARS-CoV-2 and highly homologous to 38 sarbecoviruses, including SARS-CoV-2 VOCs [157]. The vaccine was successful at eliciting SARS-CoV-2 Spike protein-specific IgG switched human B cells with a single injection adjuvanted with polyinosinic-polycytidylic acid [157]. There was also evidence of inducing human B-cell and T-cell responses in the humanized mice. Studies in macaques demonstrated a “blockade” of new cell infections, the destruction of infected cells, and improved protection against SARS-CoV-2 reinfection [157,158] without the need for an adjuvant. Currently, a combined Phase 1 and 2 trial has been registered to test this vaccine, with and without an adjuvant. They aim to recruit 240 people.

***CalTech:*** The California Institute of Technology (CalTech) is developing a Mosaic vaccine utilizing nanoparticles that display 60 randomly organized spike receptor-binding domains (RBDs) originating from the Spike trimers of eight distinct sarbecoviruses (mosaic-8 RBD nanoparticles). The Mosaic-8b vaccine is designed to produce antibodies targeting conserved and relatively hidden epitopes, as opposed to the more commonly targeted, variable, and prominently exposed epitopes. This vaccine includes RBDs from eight SARS-like betacoronaviruses (sarbecoviruses) that encompass the RBD from the currently prevalent SARS-CoV-2 virus along with RBDs from seven other animal sarbecoviruses. This diverse RBD representation is intended to provide broad-spectrum protection against a variety of related viruses. Preclinical studies have demonstrated that immunization with mosaic-8 nanoparticles elicited anti-coronavirus antibodies with robust neutralizing capabilities and effectiveness against multiple SARS-CoV-2 variants, including the highly transmissible Omicron variant. Furthermore, the vaccination conferred protection against both SARS-CoV-2 and SARS-CoV infections in mice and nonhuman primates [153,167].

***DIOSynVax:*** Cambridge University spin-off DIOSynVax (Digitally Immune Optimized Synthetic Vaccines) is utilizing a highly advanced approach in vaccine development. In collaboration with PharmaJet, they have introduced pEVAC-PS, a needle-free intradermal vaccine encoding their synthetic antigen T2-17, designed based on coronavirus receptor binding domain (RBD) sequences [168]. DIOSynVax employs a viral-genome-informed methodology to generate an antigen sequence that phylogenetically aligns with representative sequences from all known sarbecoviruses, ensuring the retention of key antibody epitopes. This synthetic antigen has been evaluated across multiple platforms, including DNA, Modified Vaccinia Ankara (MVA), and mRNA. The antigen has elicited cross-reactive neutralizing antibodies against Delta and Omicron and other tested sarbecoviruses in preclinical studies of different animal models [168].

***VBI Vaccines:*** Based on their proprietary enveloped virus-like particle (eVLP) technology, VBI Vaccines is developing vaccines that stimulate innate immune responses to viruses. Using this technology, VBI developed VBI-2901, a vaccine expressing modified prefusion forms of spike proteins from SARS-CoV-2, SARS-CoV-1, and MERS-CoV. Preclinical studies showed that VBI-2901 effectively neutralized all variants of RaTG13, including Bat RaTG13. Compared with VBI-2902, which contains only the Wuhan-Hu-1 spike protein, VBI-2901 elicited a 2.5-fold stronger response against the ancestral strain and a ninefold stronger response against the bat Coronavirus [169]. Currently, VBI-2901 is undergoing Phase I clinical trials.

***Walter Reed Army Institute of Research:*** The Walter Reed Army Institute of Research (WRAIR) has engineered a recombinant spike ferritin nanoparticle (SpFN) vaccine for SARS-CoV-2 WA-1, in combination with the Army Liposomal Formulation (ALFQ) adjuvant, which includes monophosphoryl lipid A and QS-21 (SpFN/ALFQ). This self-assembling ferritin nanoparticle is designed to present eight prefusion-stabilized SARS-CoV-2 WA-1 spike glycoprotein trimers in an ordered and symmetrical fashion. The SpFN vaccine is paired with a unilamellar liposomal adjuvant that incorporates monophosphoryl lipid A and the saponin QS-21 (ALFQ), reputed for its ability to enhance the immunogenicity of various protein vaccine candidates and its favorable tolerance in human trials. This immunogen-adjuvant combination has demonstrated the capacity to elicit broad immunity against sarbecoviruses and confer protection against SARS-CoV-2 in preclinical models [170].

### 2.6. Multifaceted Approaches to the Current State of Antigen Delivery Platforms

The development of a pancoronavirus vaccine, capable of providing broad protection against multiple coronavirus strains, requires an innovative antigen delivery approach that integrates a multi-step and multi-scale protocols [171]. This process leverages the synergy of computational and experimental techniques, enabling rapid and efficient design and optimization of antigen delivery systems [171]. This includes (1) Genomic and Proteomic Analysis: This first step involves comprehensive genomic and proteomic analysis of various coronavirus strains. Advanced bioinformatics tools employed to analyze viral genomes, identifying conserved regions across different strains that could serve as potential vaccine targets. Programs such as BLAST and Clustal Omega can be used for sequence alignment and comparison, facilitating the identification of conserved epitopes. Computational tools like AlphaFold have been used to predict conserved protein structures, while immunoinformatics platforms have identified cross-reactive epitopes for pan-Coronavirus vaccine candidates [172,173,174]. Together, these genomic and proteomic tools accelerate the rational design of vaccines that target conserved features, offering the potential for broad-spectrum protection against current and emerging coronavirus variants [174]; (2) Structural Modeling and Epitope Prediction: Following sequence analysis, structural modeling programs like Rosetta and MODELLER predict the three-dimensional structures of viral proteins. This structural data is crucial for identifying surface-exposed and accessible epitopes. Epitope prediction tools, such as BepiPred and IEDB, can be integrated to pinpoint regions likely to elicit a strong immune response. For instance, studies have used these tools to map conserved epitopes in the receptor-binding domain (RBD) of the spike protein, which are critical for neutralizing antibody responses [175,176]. By integrating structural and immunoinformatics data, these tools accelerate the rational design of vaccines with the potential to provide protection against current and emerging coronavirus variants [89]; (3) In Silico Vaccine Design: Using the identified epitopes, in silico vaccine design are performed by using tools such as VaxiJen and VaccineCAD to help in designing vaccine constructs that incorporate multiple epitopes, optimizing for immunogenicity and stability. Sequence analysis tools form the foundation of in silico vaccine design. BLAST and CLUSTAL-W facilitate the identification and alignment of conserved sequences across different coronavirus strains, enabling researchers to identify potential vaccine targets. NetMHC and the Immune Epitope Database (IEDB) [177,178,179]; (4): Molecular dynamics (MD) simulations, using platforms such as GROMACS or AMBER, provide insights into the stability and behavior of vaccine candidates at the atomic level. These simulations can predict how vaccine constructs interact with immune receptors, allowing for the refinement of vaccines designed to enhance efficacy [180,181]. MD simulations also facilitate structure-based vaccine development by enabling the analysis of protein-protein docking, antigen presentation, and the optimization of immunogenic responses. For example, studies have used MD simulations to evaluate the conformational changes in the SARS-CoV-2 spike protein and identify conserved regions that are less prone to mutations, making them ideal targets for pan-Coronavirus vaccines. Furthermore, these simulations allow for the prediction of protein stability under various environmental conditions, ensuring that vaccine candidates are both effective and stable during storage and distribution [182]; (5) In Vitro and In Vivo Validation: The most promising vaccine candidates are then synthesized and subjected to in vitro testing to assess their immunogenic properties. Techniques such as ELISA and flow cytometry are used to evaluate the immune response. Following successful in vitro results, in vivo studies in animal models further validate the vaccine’s protective efficacy; and (6) Optimization and Scale-Up: The final step involves optimizing vaccine production for scalability. Techniques such as high-throughput screening and bioreactor optimization ensure that the vaccine can be produced efficiently and at a scale suitable for widespread distribution. Programs like DOE (Design of Experiments) can help in optimizing production parameters. By integrating these multi-step and multi-scale approaches, researchers can significantly accelerate the discovery and optimization of antigen delivery systems to deliver future pan-Coronavirus vaccines. This protocol not only enhances the speed of vaccine development but also ensures a robust and comprehensive strategy to tackle current and future Coronavirus threats.

## 3. Challenges and Future Directions

While each vaccine delivery platform offers certain advantages, they also face various challenges, such as ensuring safety, manufacturing speed and scalability, vaccine stability, enhancing the breadth and duration of immune protection, and overcoming pre-existing immunity (Table 3). As research and development advances, combining attributes of these platforms may offer paths to even more effective pan-Coronavirus vaccines. The continued evolution of these platforms in the context of pan-Coronavirus vaccine development is a testament to the rapid progress being made in immunology and virology, offering hope for durable, broad-spectrum protection against current and emergent coronavirus threats.

Although the SAPNs platform has been extensively studied since 2006 and was successful in pre-clinical animal studies, as reported for a preventive malaria vaccine [90,98], there are no reported successful clinical trials against Malaria, or any other infectious diseases, including SARS-CoV-2, using this particular SAPNs technology [90,98]. Thus, although a SAPNs-based vaccine candidate appears immunogenic in a mouse model [90,98], to the best of our knowledge, a subsequent SAPNs-based malaria-vaccine clinical trial appears to have been terminated. There are no reported peer-reviewed animal or human studies demonstrating the superiority of this SAPN platform [90,98], even when combined with an adjuvants (e.g., Flagellin and/or CpG) compared to the recently discovered mRNA/LNP platform when it comes to safety, immunogenicity, and protective efficacy against any infectious diseases, including SARS-CoV-2. Thus, the mRNA/LNP delivery system, which won the 2023 Nobel Prize of Medicine, remains the best antigen delivery system to delivery future pan-Coronavirus vaccines.

## 4. Conclusions

Pan-Coronavirus vaccines represent a potentially bold step forward in the global fight against current and emerging coronavirus infectious diseases. While significant challenges remain, the advancement of various pan-Coronavirus vaccine candidates employing various antigen delivery systems towards clinical trials would offer the opportunity to better understand the critical attributes needed for an effective antigen delivery platforms for Next-Generation Pan-Coronavirus vaccine. The ultimate success of these pan-Coronavirus vaccines will depend not only on their clinical safety and efficacy but also on the ease with which these vaccines can be manufactured and distributed globally at a large scale. As the pan-Coronavirus vaccine clinical trial landscape evolves, so too will our hope for future resilience to coronavirus outbreaks. 

## Figures and Tables

**Table 1:** Characteristics of different types of mRNA used to deliver next-generation pan-Coronavirus vaccine candidates.

Type of RNA Vaccine	Size of mRNA	Half-Life in Cells	Expression Duration	Cold Chain Required?	Mode of Action	Example of Vaccine	Sources
Conventional and base-modified mRNA, non-amplifying mRNA	Variable typically 1–5 kb	6–12 h	1–3 days	Yes, typically requires cold storage (refrigeration)	Uses chemically modified nucleotides to enhance stability and translation, leading to protein antigen production and immune response induction.	BioNTech/Pfizer. Highly effective, 95% efficacy, approved globally. Moderna. Highly effective, 94% efficacy, approved globally.	[55,56]
Self-amplifying mRNA (saRNA)	10–15 kb	2–4 days	1–2 months	Yes, typically requires cold storage (refrigeration)	Contains viral replicase machinery that allows the RNA to self-amplify within cells, producing a larger amount of antigen over an extended period.	Arcturus Therapeutics. Ongoing, early results show strong immune response. Imperial College London. Phase 1 trial ongoing, safety and immune response being evaluated Gritstone. Phase I trials ongoing.	[51,57,58]
Circular mRNA (circRNA)	Variable, typically 1–5 kb	1–3 weeks	1–3 months	No, can be stored at room temperature	Circular structure is resistant to exonucleases, leading to increased stability and prolonged antigen expression.	Laronde. Preclinical studies indicate potential for durability and stability	[52,59]
Thermostable mRNA	Variable typically 1–5 kb	6–12 h	1–3 days	No, can be stored at room temperature	Engineered to maintain stability and function at higher temperatures.	CureVac. Moderate efficacy, 48% in Phase 3 trials	[60,61]

**Table 2:** Current pan-Coronavirus vaccines in different stages of clinical trials.

Vaccine Name	Vaccine Type	Developer	Clinical Phase	Dose and Regimen	Antigen	Publications (PMID, Year)
BNT162b4	mRNA	Pfizer	Phase I trial ongoing	N/A	Segments of the SARS-CoV-2 nucleocapsid, membrane, and ORF1ab proteins, targeting diverse HLA alleles.	[151]
GRT-R910	saRNA	Gritstone bio, Inc.	Phase 1, NCT05148962	two different doses (10 or 30 mcg)	Full-length Spike and selected conserved non-Spike T cell epitopes.	[58]
Mosaic-8b	Protein subunit	California Institute of Technology (Caltech)	Yet to begin	N/A	1 SARS-CoV-2 RBD + 7 sarbecovirus RBD	[26,152,153,154,155]
DIOS-CoVax/ pEVAC-PS	mRNA	DIOSynvax	Phase I, (https://www.isrctn.com/ISRCTN87813400, (accessed on 1 January 2024)	1.2 mg, 0.8 mg 0.4 mg, or 0.2 mg, two doses, 30 days apart	T2_17 (DIOSynVax Generated)	[26]
CD40.CoV2	Protein subunit	Inserm Vaccine Research Institute	Yet to begin	N/A	Receptor-binding domain (RBD) of the SARS-CoV-2 spike protein	[26,156,157,158]
OVX033	Protein subunit	Osivax	Yet to begin	N/A	Nucleocapsid (N) Protein	[159]
Unnamed	mRNA, Viral vector and Protein subunit	University of California Irvine/TechImmune LLC	Yet to begin	N/A	Multi-epitope	[1,80]
VBI-2901	eVLP	VBI Vaccines	Phase I, NCT05548439	15–20 µg, two doses, 55 days apart	Spike trivalent (SARS- CoV-1, SARS-CoV-2, MERS-CoV)	[26]
SpFN 1B-06-PL	Protein subunit	Walter Reed Army Institute of Research (WRAIR)	Phase I, NCT04784767	25 µg or 50 µg, two doses	SARS-CoV-2 Spike Ferritin Nanoparticle	[160,161,162,163,164,165,166]

**Table 3:** Characteristics of different platform types used to deliver next-generation pan-Coronavirus vaccine candidates.

Pancoronavirus Vaccine Platforms Type	Mechanism of action	Advantages	Disadvantages	Challenges	Examples
mRNA Vaccines	Use mRNA to instruct cells to produce viral proteins, eliciting an immune response	-High effectiveness in protein translation -High immunogenicity and flexibility to adapt to new variants -Non-infectious, no risk of genome integration -Rapid development and production	-Requires ultra-cold storage -antigen expression duration is limited -complicates encapsulation/delivery and manufacturing -Potential for higher reactogenicity	-Ensuring global cold chain logistics -Addressing public concerns and vaccine hesitancy	-Pfizer BNT162b4 Pfizer -From—Gritstone GRT-R910: GRT-R910 from Gritstone
Viral Vector Vaccines	Use a harmless virus to deliver genetic material coding for viral proteins, inducing an immune response	-Elicit strong immune responses -Potential for single-dose immunization	-Pre-existing immunity to the vector can reduce efficacy -More complex manufacturing processes	-Balancing efficacy with safety, especially vector-related -Efficiently scaling up production	-AZD1222 vaccine from AstraZeneca and the University of Oxford -Covishield from Serum Institute of India
Protein Subunit Vaccines	Introduce purified viral proteins subunits directly to stimulate an immune response	-Established technology with a good safety profile -Stable at standard refrigeration temperatures -Non-infectious, no risk of genome integration	-Often require adjuvants to enhance immune response -Often need multiple doses	-Achieving desired immunogenicity without compromising safety -Ensuring cost-effective mass production	-OVX033 from Osivax—Mosaic-8b from CalTech -SpFN 1B-06-PL from Walter Reed Army Institute of Re-search (WRAIR)
